# Complete Genome Sequence of a Newcastle Disease Virus Isolate from an Outbreak in Central India

**DOI:** 10.1128/genomeA.01418-14

**Published:** 2015-01-15

**Authors:** Polakshee Gogoi, Sudhir Morla, Megha Kaore, Nitin Vasantrao Kurkure, Sachin Kumar

**Affiliations:** a Department of Biotechnology, Indian Institute of Technology Guwahati, Guwahati, Assam, India; b Department of Pathology, Nagpur Veterinary College, Maharashtra Animal & Fishery Sciences University, Nagpur, India

## Abstract

The complete genome sequence of a Newcastle disease virus (NDV) strain NDV/Chicken/Nagpur/01/12 was isolated from vaccinated chicken farms in India during outbreaks in 2012. The genome is 15,192 nucleotides in length and is classified as genotype VII in class II.

## GENOME ANNOUNCEMENT

Newcastle disease virus (NDV) causes a highly pathogenic viral disease known as Newcastle disease (ND) in avian species. It is classified under avian paramyxovirus serotype 1 (APMV-1). NDV is a single-stranded, nonsegmented, negative sense RNA virus belonging to the genus *Avulavirus*, in the family *Paramyxoviridae* (1). The genome sizes of NDV strains isolated from different parts of the world are categorized into three different groups: the isolates before 1960 are 15,186 nucleotides (nt) long, the isolates reported from China are 15,192 nt long, and the isolates discovered in Germany are 15,198 nt long (2). Based on genetic analysis, strains of NDV are classified into classes I and II. Avirulent strains isolated from wild birds are classified under class I genotype, whereas both virulent and avirulent strains isolated from wild and domestic birds are classified under class II (3).

In India, there have been continuous reports of ND outbreaks which have affected the economy of domestic poultry industries significantly despite various drives of vaccination programs (4, 5). For effective control of NDV, a greater understanding of NDV’s genetic diversity is necessary. In the year 2012, ND outbreaks were reported from chicken farms located in the Nagpur district of the state Maharashtra in India. The affected commercial chickens were vaccinated with NDV strain LaSota. The outbreak has caused nearly 100% mortality. The various pathognomonic signs and symptoms observed in the diseased chickens were pinpoint hemorrhages at the tips of the proventricular glands and cecal tonsil and button ulcers in the duodenum and large intestine. In addition, mild congestion of brain, liver, and in the bursa was also observed. The virus was isolated and propagated in 9-day-old embryonated chicken eggs. One of the isolates, namely, NDV/Chicken/Nagpur/01/12, was plaque purified in chicken embryo fibroblast and the complete genome sequence was determined by reverse transcription (RT)-PCR using overlapped consensus primers and direct sequencing (6). To determine the 3′ and 5′ ends of the viral genome, rapid amplification of cDNA ends (RACE) was used (7). The complete genome of NDV/Chicken/Nagpur/01/12 is 15,192 in length. The sequence identities of amino acids of fusion (F) and hemagglutinin-neuraminidase (HN) proteins between NDV strain NDV/Chicken/Nagpur/01/12 and the lentogenic vaccine strain LaSota are 88.4% and 85.3%, respectively. From the sequence analysis it has been found that the circulating strains of the outbreak are distinctly different from the vaccine strain.

The sequence of the F protein cleavage site is the major determinant of the NDV pathogenicity. The strain NDV/Chicken/Nagpur/01/12 has a virulent pathotype, with ^112^RRQKR^116^ at the C terminus of the F2 protein and F at residue 117 (8). The phylogenetic analysis by MEGA 5.2 of the F gene suggests that the present isolate belongs to genotype VII of class II (9).

### Nucleotide sequence accession number.

The complete genome sequence of NDV/Chicken/Nagpur/01/12 is deposited in GenBank under the accession no. KP089979.

## References

[1] Lamb R, Parks G 2007 Paramyxoviridae: the viruses and their replication, p 1449–1496. *In* Knipe DM, Howley PM, Griffin DE, Lamb RA, Martin MA, Roizman B, Straus SE (ed), Fields Virology, 5th ed. Lippincott Williams & Wilkins, Philadelphia, PA.

[2] Czeglédi A, Ujvári D, Somogyi E, Wehmann E, Werner O, Lomniczi B 2006 Third genome size category of avian paramyxovirus serotype 1 (Newcastle disease virus) and evolutionary implications. Virus Res 120:36–48. doi:10.1016/j.virusres.2005.11.009. 16766077

[3] Miller PJ, Decanini EL, Afonso CL 2010 Newcastle disease: evolution of genotypes and the related diagnostic challenges. Infect Genet Evol 10:26–35. doi:10.1016/j.meegid.2009.09.012. 19800028

[4] Ganar K, Das M, Sinha S, Kumar S 2014 Newcastle disease virus: current status and our understanding. Virus Res 184:71–81. doi:10.1016/j.virusres.2014.02.016. 24589707 PMC7127793

[5] Morla S, Kumar Tiwari A, Joshi V, Kumar S 2014 Complete genome sequence of a Newcastle disease virus isolate from an outbreak in northern India. Genome Announc 2(2):e00342-14. doi:10.1128/genomeA.00342-14. 24762939 PMC3999496

[6] Paldurai A, Kumar S, Nayak B, Samal SK 2010 Complete genome sequence of highly virulent neurotropic Newcastle disease virus strain Texas GB. Virus Genes 41:67–72. doi:10.1007/s11262-010-0486-3. 20431932

[7] Kumar S, Nayak B, Collins PL, Samal SK 2008 Complete genome sequence of avian paramyxovirus type 3 reveals an unusually long trailer region. Virus Res 137:189–197. doi:10.1016/j.virusres.2008.07.012. 18691616 PMC2666097

[8] Panda A, Huang Z, Elankumaran S, Rockemann DD, Samal SK 2004 Role of fusion protein cleavage site in the virulence of Newcastle disease virus. Microb Pathog 36:1–10. doi:10.1016/j.micpath.2003.07.003. 14643634 PMC7125746

[9] Diel DG, da Silva LH, Liu H, Wang Z, Miller PJ, Afonso CL 2012 Genetic diversity of avian paramyxovirus type 1: proposal for a unified nomenclature and classification system of Newcastle disease virus genotypes. Infect Genet Evol 12:1770–1779. doi:10.1016/j.meegid.2012.07.012. 22892200

